# The Effect of Video Game–Based Interventions on Performance and Cognitive Function in Older Adults: Bayesian Network Meta-analysis

**DOI:** 10.2196/27058

**Published:** 2021-12-30

**Authors:** Chao Yang, Xiaolei Han, Mingxue Jin, Jianhui Xu, Yiren Wang, Yajun Zhang, Chonglong Xu, Yingshi Zhang, Enshi Jin, Chengzhe Piao

**Affiliations:** 1 Department of Ethnic Culture and Vocational Education Liaoning National Normal College Shenyang China; 2 Department of Clinical Pharmacy Shenyang Pharmaceutical University Shenyang China; 3 Chi Chi Technology LLC Shenyang China; 4 Information Construction Department Liaoning National Normal College Shenyang China

**Keywords:** video game, performance, cognitive function, older, Bayesian network meta-analysis

## Abstract

**Background:**

The decline in performance of older people includes balance function, physical function, and fear of falling and depression. General cognitive function decline is described in terms of processing speed, working memory, attention, and executive functioning, and video game interventions may be effective.

**Objective:**

This study evaluates the effect of video game interventions on performance and cognitive function in older participants in terms of 6 indicators: balance function, executive function, general cognitive function, physical function, processing speed, and fear of falling and depression.

**Methods:**

Electronic databases were searched for studies from inception to June 30, 2020. Randomized controlled trials and case-controlled trials comparing video game interventions versus nonvideo game control in terms of performance and cognitive function outcomes were incorporated into a Bayesian network meta-analysis. All data were continuous variables.

**Results:**

In total, 47 studies (3244 participants) were included. In pairwise meta-analysis, compared with nonvideo game control, video game interventions improved processing speed, general cognitive function, and depression scores. In the Bayesian network meta-analysis, interventions with video games improved balance function time (standardized mean difference [SMD] –3.34, 95% credible interval [CrI] –5.54 to –2.56), the cognitive function score (SMD 1.23, 95% CrI 0.82-1.86), processing speed time (SMD –0.29, 95% CrI –0.49 to –0.08), and processing speed number (SMD 0.72, 95% CrI 0.36-1.09), similar to the pairwise meta-analysis. Interventions with video games with strong visual senses and good interactivity ranked first, and these might be more beneficial for the elderly.

**Conclusions:**

Our comprehensive Bayesian network meta-analysis provides evidence that video game interventions could be considered for the elderly for improving performance and cognitive function, especially general cognitive scores and processing speed. Games with better interactivity and visual stimulation have better curative effects. Based on the available evidence, we recommend video game interventions for the elderly.

**Trial Registration:**

PROSPERO CRD42020197158; https://www.crd.york.ac.uk/prospero/display_record.php?RecordID=197158

## Introduction

### Background

Since the mid-20th century, with steady improvements in living standards and the growing affluence of global societies, greater longevity has led to great concern over the decline in performance and cognitive abilities that accompanies normal and neuropathological aging [1]. The decline in performance includes balance function, physical function, and fear of falling and depression. General cognitive function decline is described in terms of processing speed, working memory, attention, and executive functioning [2-5]. These demographic changes and neurological aging compromise the sustainability of health care systems, with more health care resources needed to care for the aging population [6]. Therefore, it is essential to propose feasible and acceptable interventions to promote active aging, intended to preserve and optimize opportunities for health, participation, and security in order to enhance the quality of life as people age.

### Objective

With the rapid development of computer technology and the video game industry, game types and game experience have greatly improved. A video game is any game played on a digital device, encompassing a wide range of interfaces, including web-based programs and apps for mobile devices [7]. Exergames are video games that require physical activity and movement when played [8]. Video games are promising and adaptable cognitive training tools in the active aging process that can help stave off the negative effects of aging in performance and cognitive function. Moreover, video games are inexpensive and interesting and can be employed in hospitals as well as in the community [9]. Furthermore, older adults are now more digitally connected than ever, and most older people aged 65 years or more have a computer with an internet connection [10]. Therefore, the use and implementation of video game interventions are high.

Although off-the-shelf video games were not developed for serious purposes or these specific interventions, many studies have determined that video game interventions may benefit the elderly in terms of serious purposes, including cognitive function, fall prevention, and other benefits [1,11,12]. No previous systematic review has provided a comprehensive overview with meta-regression and Bayesian network meta-analysis evaluating which type of video game intervention has the best effect on performance and cognitive function.

## Methods

This systematic review conformed to PRISMA (Preferred Reporting Items for Systematic Reviews and Meta-Analysis) and the PRISMA extension statement for network meta-analysis [13,14]. We registered the protocol for this Bayesian network meta-analysis with the International Prospective Register of Systematic Reviews (PROSPERO; CRD42020197158) [15].

### Search Strategy and Selection Criteria

We conducted systematic literature searches of PubMed, EMBASE, and the Cochrane Library from their inception to June 30, 2020. The MeSH search terms were as follows in the full-text search: “active video game,” “active game,” “older adults,” and “elderly.” We included both randomized controlled trials (RCTs) and case-controlled trials (CCTs) that met the following criteria: participants were older adults (aged ≥60 years) without dementia; the interventions were all kinds of active video games (such as those on Xbox 360 or Nintendo Wii, computer-based games, virtual reality–based games), which means that the participants perform the video game intervention in an active state rather than a static state, and the whole body needs to be involved in the video games; and controls included exercise, puzzle games, visual stimulation, and no game play, which means that even patients who play games are still in a static state. The above declared the difference between the intervention group and the control group in order to study the effect of active video games in the intervention group. Comparisons of interventions with video games versus nonvideo game control were made with reported outcomes of performance and cognitive function.

### Outcome Assessment

We assessed the effectiveness of 6 performance and cognitive outcomes by comparing the intervention group and the control group at the final point. The first outcome was balance function, tested using the Berg Balance Scale (BBS), balance test time (s), and balance test speed (m/s). The second outcome was executive function, tested using performance on the trail-making test B (TMT-B, s), delta (s), Stroop word (s), attention, working memory, and the Corsi block test. The third outcome was related to general cognition, tested using the Mini-Mental State Exam (MMSE; score) and the Montreal Cognitive Assessment (MoCA; score). Physical function was the fourth outcome, tested using everyday function and function tests (s and cm). The fifth outcome was processing speed tested using the TMT-A (s) and processing speed (number). The final outcomes were the fear of falling and depression, tested using the Efficacy Scale International (score) and the Geriatric Depression Scale (score). All these outcomes were continuous data.

### Data Extraction and Quality Assessment

Two investigators (CY and XH) independently conducted the electronic literature search. The reference lists of relevant publications were also checked, and no language restrictions were set. These 2 researchers evaluated eligible titles, abstracts, and full texts, and disagreements between them were resolved by discussion with a third researcher (author YZ [Yingshi Zhang] or PC). A preset table was designed to extract details of potentially relevant papers, including the first author, publishing year, study type, region, sample size, gender, age, body mass index (BMI), education year, MMSE score, video game type, frequency, period, follow-up, control type, other associated diseases, and community or hospital. Two investigators (authors CY and EJ) extracted all continuous data independently onto a Microsoft Excel spreadsheet. The quality of the included RCTs was evaluated according to Cochrane Collaboration’s tool for assessing the risk of bias [16], and the quality of the included CCTs was assessed using the Newcastle-Ottawa Scale (NOS) score [17]. We also used the Grading of Recommendations, Assessment, Development and Evaluations (GRADE) scale [18] to evaluate the quality of outcomes. Disagreements were resolved by a third researcher (YZ [Yingshi Zhang] or PC).

### Data Synthesis and Statistical Analysis

We applied standardized mean differences (SMDs) and 95% CIs to summarize the 6 outcomes from pairwise meta-analysis. To determine the efficacy of video game intervention versus nonvideo game control, we performed subgroup analysis and meta-regression of various intervention types (ie, Nintendo Wii, Xbox 360, and other video games), types of activity control activities (eg, physical activity, visual stimulation, puzzle games, and no game play), and periods (0-4 weeks, 4-8 weeks, 8-12 weeks, and more than 12 weeks). To determine the heterogeneity among our included studies, *P*≤.05 or I^2^>50% indicated heterogeneity in the outcome. *P*<.10 revealed that a grouping method was a source of heterogeneity. The random effects model was used to ensure the accuracy of the summarized data. Publication bias was assessed using the Begg and Egger tests, where *P*≤.05 indicated the existence of publication bias [19,20].

We performed a Bayesian random effects network meta-analysis composed of 4 chains with 100,000 iterations after an initial burnin of 10,000 and a thinning of 2.5 in order to determine the most suitable video game intervention. We calculated the SMDs and corresponding 95% credible intervals (CrIs), and the mean rank and surface under the cumulative ranking curve (SUCRA) values were produced from network meta-analysis estimates with a consistent model. We also produced comparison-adjusted funnel plots to explore publication bias from network meta-analysis. All the aforementioned analyses were performed using StataMP, version 14.0 (StataCorp) and WinBUGS, version 1.4.3 (MRC Biostatistics Unit and Imperial College School of Medicine).

## Results

### Description of Included Studies

**Figure 1:**
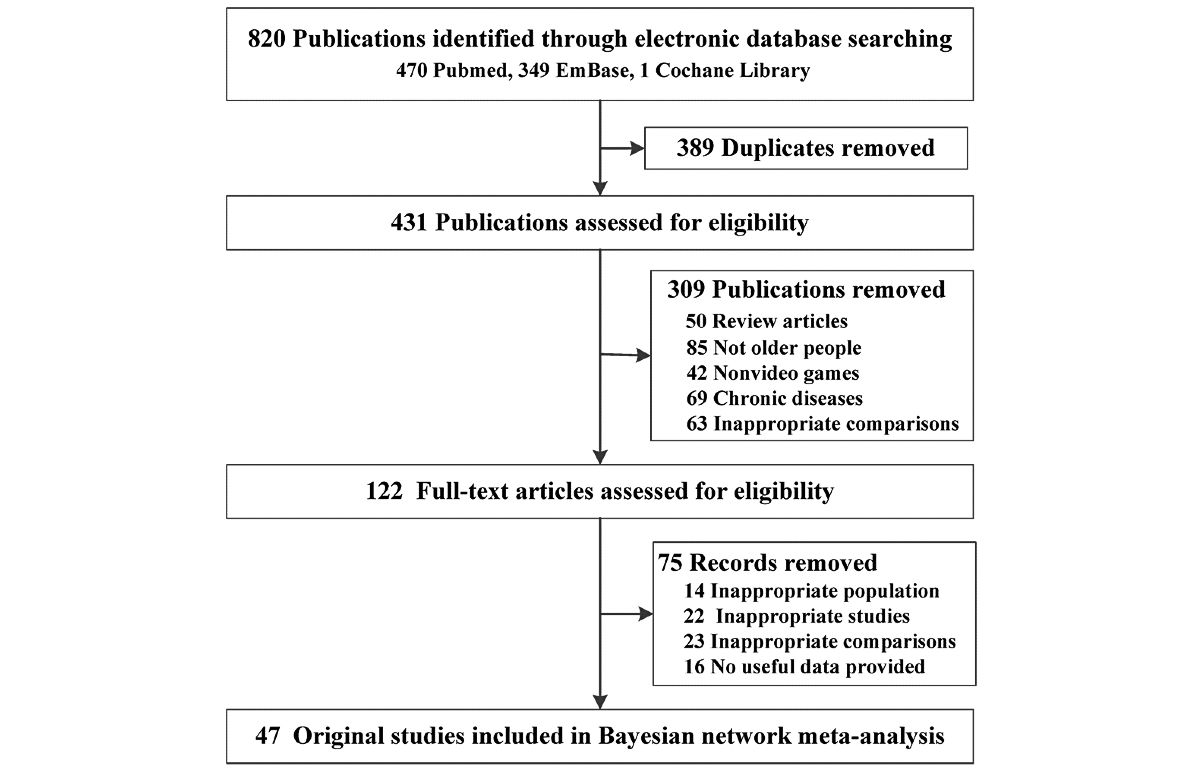
Study selection flowchart.

Figure 1 shows details of the selection process. The search strategy generated 820 citations in total. After duplication removal and preliminary screening, 122 potentially publications were scrutinized for eligibility. Finally, we identified 47 original studies [21-66] that met the inclusion criteria. Only 1 (2.1%) of them was a CCT, and the remaining 46 (97.9%) were RCTs. Overall, 1651 of 3244 (50.9%) participants were assigned to the intervention group and the remaining 1593 (49.1%) were assigned to the control group. The sample sizes ranged from 12 to 977 (Table 1 and Multimedia Appendix 1). Baseline characteristics were balanced except that the intervention group was notably older (Table 2). Risk-of-bias assessment was performed for each RCT and CCT, and all included studies had acceptable quality (Multimedia Appendices 2 and 3).

**Table 1:** Summarized baseline characteristics of included studies.

Control type		Source, year	Study type	Sample size (I/C)^a^
**Intervention type: Xbox 360**				
	No game play	Rica et al, 2020 [21]	RCT^b^	25/25
	No game play	Sápi et al, 2019 [29]	RCT	30/22
	No game play	Sato et al, 2015 [51]	RCT	28/26
	Normal exercises of upper and lower limbs	Amjad et al, 2019 [22]	RCT	20/18
	Adventures and sports	Sápi et al, 2019 [29]	RCT	30/23
	Conventional physical therapy	Bacha et al, 2018 [32]	RCT	23/23
	Home exercise group	Karahan et al, 2015 [49]	RCT	48/42
**Intervention type: Nintendo Wii**				
	Tai chi chuan	Gatica-Rojas et al, 2019 [25]	CCT	7/5
	Traditional exercise	Li et al, 2018 [34]	RCT	49/53
	No game play	Montero-Alía et al, 2019 [27]	RCT	508/469
	No game play	Zadro et al, 2019 [31]	RCT	30/30
	No game play	Gomes et al, 2018 [33]	RCT	15/15
	No game play	Franco et al, 2012 [59]	RCT	11/10
	No game play	Maillot et al, 2012 [60]	RCT	15/15
	No game play	Singh et al, 2012 [64]	RCT	18/18
	Fall prevention education	Lee et al, 2017 [37]	RCT	21/19
	Same movements	Monteiro-Junior et al, 2017 [38]	RCT	10/9
	Gym exercise class	Kwok et al, 2016 [42]	RCT	40/40
	Ethylene vinyl acetate copolymer insoles	Jorgensen et al, 2013 [55]	RCT	28/30
	Therapeutic balance exercise group	Singh et al, 2013 [57]	RCT	18/18
	Seated exercise group	Daniel et al, 2012 [58]	RCT	8/8
	Physical activity	Daniel et al, 2012 [58]	RCT	8/7
	Completed exercises	Franco et al, 2012 [59]	RCT	11/11
**Intervention type: video game training**				
	Insight process-based intervention	Belchior et al, 2019 [23]	RCT	17/19
	No game play	Belchior et al, 2019 [23]	RCT	17/18
	No game play	Sosa et al, 2019 [30]	RCT	20/15
	No game play	Ordnung et al, 2017 [39]	RCT	14/15
	No game play	Toril et al, 2016 [45]	RCT	19/20
	No game play	Schoene et al, 2015 [56]	RCT	47/43
	No game play	Belchior et al, 2013 [54]	RCT	14/13
	No game play	Pichierri et al, 2012 [62]	RCT	11/11
	Simulation strategy games	Szelag et al, 2018 [35]	RCT	30/25
	Visual stimulation	Buitenweg et al, 2017 [36]	RCT	56+33/50
	Common puzzle games	Souders et al, 2017 [40]	RCT	30/30
	Balance and stretching training	Eggenberger et al, 2016 [41]	RCT	19/14
	Balance and stretching training	Schättin et al, 2016 [44]	RCT	13/14
	Knowledge quiz training game	Nouchi et al, 2016 [43]	RCT	36/36
	Knowledge quiz training game	van Muijden et al, 2012 [65]	RCT	53/19
	Education booklet	Gschwind et al, 2015 [48]	RCT	78/75
	Community center–based activities	Kim et al, 2015 [50]	RCT	14/14
	Physical activity	Whyatt et al, 2015 [53]	RCT	40/42
	Placebo training condition	Belchior et al, 2013 [54]	RCT	14/15
	Placebo training condition	Nouchi et al, 2012 [61]	RCT	14/14
	Usual activities	Schoene et al, 2013 [56]	RCT	15/17
**Intervention type: computer-based games**				
	No game play	Faust et al, 2019 [24]	RCT	25/19
	No game play	Perrot et al, 2019 [28]	RCT	12/11
	Kawashima brain training	Perrot et al, 2019 [28]	RCT	12/12
	Met researchers	Ballesteros et al, 2014 [35]	RCT	17/13
	Typical rehabilitation program	Szturm et al, 2011 [66]	RCT	13/14
**Intervention type: virtual reality–based games**				
	Combined physical and cognitive training	Liao et al, 2019 [26]	RCT	18/16
	Conventional exercise	Yeşilyaprak et al, 2016 [46]	RCT	7/11
	Treadmill memory training	Eggenberger et al, 2015 [47]	RCT	24/22
	Treadmill walking	Eggenberger et al, 2015 [47]	RCT	24/25
	No game play	Rendon et al, 2012 [63]	RCT	20/20

^a^I: intervention group; C: control group.

^b^RCT: randomized controlled trial.

**Table 2:** Balance status of baseline characteristics (italics indicate a significant difference).

Baseline indicator	SMD^a^/OR^b^ (95% CI)	*P* value	I^2^ (%)	Baseline balanced
Age	*0.236 (0.018-0.454)*	<.001	87.1	No
Gender	0.939 (0.757-1.163)	.42	2.9	Yes
Body mass index	–0.113 (–0.311 to 0.084)	<.001	62.6	Yes
Education	0.026 (–0.161 to 0.213)	.24	19.1	Yes
Mini-Mental State Exam	0.023 (–0.172 to 0.217)	.19	25.2	Yes

^a^SMD: standardized mean difference.

^b^OR: odds ratio.

### Outcomes of Pairwise Meta-analysis

For the outcomes of balance function, we first compared balance scores (BBS, etc) pre- and postintervention. No significant differences were found overall and in subgroups by intervention and control types. Significant differences were only found at 8-12 weeks. Substantial heterogeneity was found overall and in all subgroups. The source of heterogeneity was not determined from meta-regression, and publication bias was occasionally found. For the balance test time (s), no significant differences were found overall and in all subgroup meta-analyses, with frequent substantial heterogeneity and no publication bias. In balance test speed (m/s) assessment, significant differences were found only for no game play as the control type (odds ratio [OR] 0.611, 95% CI 0.048-1.175). These findings suggest that video game interventions may improve balance in the elderly (Table 3); however, the most suitable intervention has not yet been determined.

For assessment of executive function, we first evaluated the results of the TMT-B (s). No significant differences were found in all subgroups, with frequent substantial heterogeneity. Second, no significant difference was found in the outcome of delta (s). Similarly, no significant differences were found in the subgroup and overall outcomes of the Stroop word test (s), with substantial heterogeneity. When we evaluated the attention in video games compared with nonvideo games, significant differences were found only in the period of more than 12 weeks, with only 1 original study. In the evaluation of working memory, significant differences were detected for overall outcomes (OR=1.034, 95% CI 0.305-1.763), other video games as the intervention type (OR=1.076, 95% CI 0.295-1.858), no game play as the control type (OR 1.023, 95% CI 0.133-1.914), and 4-8 weeks as the intervention period (OR 1.401, 95% CI 0.559-2.243), with substantial moderate-grade heterogeneity in these subgroups. For the Corsi block test, significant differences were found for no game play and intervention periods of 0-4 and 8-12 weeks. Only the outcome for 8-12 weeks in 4 studies showed no heterogeneity (OR 0.429, 95% CI 0.080-0.778, *P=*.42, I^2^=0.0%) with high quality (Table 4). In summary, video game interventions had little effect on executive function, except for memory-related functions.

**Table 3:** Summarized outcomes from pairwise meta-analysis of performance and cognitive data for balance function (italics indicate a significant difference).

Variable		Subgroup	Studies (n)	OR^a^ (95% CI)	*P* value, I^2^	Meta-regression	Grade	Publication bias, *P* value
**BBS^b^**								
	Overall	N/A^c^	26	0.213 (–0.025 to 0.451)	<.001, 83.5%^d^	0.362	Moderate	0.499, .02
	Intervention type	Nintendo Wii	14	0.187 (–0.059 to 0.432)	<.001, 74.0%^d^	—^e^	Moderate	0.547, .02
	Intervention type	Xbox 360	3	0.419 (–0.133 to 0.971)	.03, 71.1%^d^	—	Low	0.602, .35
	Intervention type	Other video games	9	0.093 (–0.664 to 0.850)	<.001, 89.7%^d^	—	Moderate	0.420, .85
	Control type	Activity control	13	0.190 (–0.290 to 0.669)	<.001, 85.5%^d^	0.812	Moderate	0.870, .46
	Control type	No game play	13	0.159 (–0.095 to 0.413)	<.001, 75.5%^d^		Moderate	0.493, .03
	Period	0-4 weeks	7	0.127 (–0.192, 0.446)	.73, 0.0%	0.902	High	0.881, .41
	Period	4-8 weeks	16	0.362 (–0.103 to 0.826)	<.001, 87.1%^d^	—	Moderate	0.719, .99
	Period	8-12 weeks	3	–*0.178 (–0.265* to *0.090)*	.73, 0.0%	—	Moderate	0.602, .31
**Balance test (s)**								
	Overall	N/A	22	–0.090 (–0.348 to 0.168)	<.001, 72.4%	—	—	0.310, .26
	Intervention type	Nintendo Wii	9	–0.364 (–0.846 to 0.119)	<.001, 78.4%^d^	0.766	Moderate	0.144, .27
	Intervention type	Xbox 360	4	0.084 (–0.613 to 0.780)	<.001, 85.7%^d^	—	Moderate	0.404, .36
	Intervention type	Other video games	9	0.125 (–0.135 to 0.385)	.23, 24.2%	—	High	1.000, .66
	Control type	Activity control	16	–0.081 (–0.317 to 0.156)	.001,59.2%^d^	0.968	Moderate	0.458, .32
	Control type	No game play	6	–0.156 (–1.128 to 0.816)	<.001, 89.1%^d^	—	Moderate	0.624, .53
	Period	0-4 weeks	1	–0.515 (–1.780 to 0.750)	—	0.565	Low	—
	Period	4-8 weeks	13	–0.105 (–0.372 to 0.162)	.02, 50.9%^d^	—	Moderate	0.903, .90
	Period	8-12 weeks	5	–0.100 (–0.923 to 0.723)	<.001, 91.2%^d^	—	Moderate	0.881, .59
	Period	More than 12 weeks	3	0.192 (-0.116 to 0.499)	.36, 2.4%	—	Moderate	—
**Balance test speed (m/s)**								
	Overall (all 4-8 weeks)	N/A	5	–0.046 (–0.970 to 0.878)	<.001, 84.5%^d^	—	Moderate	0.355, <.001
	Intervention type	Nintendo Wii	3	–1.101 (–3.627 to 1.426)	<.001, 91.9%^d^	0.551	Moderate	0.117, .03
	Intervention type	Xbox 360	2	0.394 (–0.021 to 0.808)	.29,10.9%	—	Moderate	—
	Control type	Activity control	4	–0.449 (–1.796 to 0.898)	<.001, 88.1%^d^	0.660	Moderate	0.174, .22
	Control type	No game play	1	*0.611 (0.048-1.175)*	—	—	Low	—

^a^OR: odds ratio.

^b^BBS: Berg Balance Scale.

^c^N/A: not applicable.

^d^Substantial heterogeneity.

^e^—: not applicable.

**Table 4:** Summarized outcomes from pairwise meta-analysis of performance and cognitive data for executive function (italics indicate a significant difference).

Variable		Subgroup	Studies (n)	OR^a^ (95% CI)	*P* value, I^2^	Meta-regression	Grade	Publication bias, *P* value
**TMT-B^b^ (s)**								
	Overall	N/A^c^	16	–0.203 (–0.807 to 0.402)	<.001,93.6%^d^	—^e^	Moderate	0.392, .75
	Intervention type	Other video games	15	0.077 (–0.461 to 0.615)	<.001, 91.9%^d^	—	Moderate	0.729, .52
	Control type	Activity control	13	–0.209 (–0.972 to 0.554)	<.001, 94.9%^d^	0.995	Moderate	0.542, .78
	Control type	No game play	3	–0.100 (–0.424 to 0.223)	.98, 0.0%	—	Moderate	0.602, .29
	Period	0–4 weeks	2	1.095 (–2.738 to 4.927)	<.001, 98.1%^d^	0.246	Low	0.317, —
	Period	4–8 weeks	5	–0.141 (–2.476 to 2.193)	<.001, 96.7%^d^	—	Moderate	0.624, .88
	Period	8–12 weeks	4	–0.083 (–0.371 to 0.205)	.52, 0.0%	—	Moderate	1.000, .22
	Period	More than 12 weeks	4	–0.684 (–1.487 to 0.119)	<.001, 91.3%^d^	—	Moderate	0.042, .03
**Delta (s)**								
	Overall	N/A	3	–1.780 (–3.758 to 0.198)	<.001, 93.8%^d^	—	Low	0.296, .12
**Stroop word (s)**								
	Overall (all other video games)	N/A	14	0.050 (–0.275 to 0.374)	<.001, 76.6%^d^	—	Moderate	0.261, .83
	Control type	Activity control	10	0.135 (–0.281 to 0.552)	<.001, 80.6%^d^	0.541	Moderate	0.245, .95
	Control type	No game play	4	–0.169 (–0.640 to 0.301)	.01, 54.7%^c^	—	Moderate	1.000, .82
	Period	0–4 weeks	1	0.530 (0.060-1.001)	—	0.669	—	0.317, —
	Period	4–8 weeks	6	0.015 (–0.787 to 0.818)	<.001, 87.2%^d^	—	Moderate	0.624, .15
	Period	8–12 weeks	5	0.039 (–0.475 to 0.554)	.02, 66.3%^d^	—	Moderate	0.327, .21
	Period	More than 12 weeks	2	–0.017 (–0.268 to 0.235)	.92,0.0%	—	Moderate	0.317, —
**Attention**								
	Overall (all other video games)	N/A	9	0.185 (–1.607 to 1.976)	<.001, 97.7%^d^	—	Moderate	0.137, .32
	Control type	Activity control	7	1.317 (–0.158 to 2.792)	<.001, 96.8%^d^	0.116	Moderate	0.099, .37
	Control type	No game play	2	–17.991 (–64.346 to 28.364)	<.001, 99.3%^d^	—	Low	0.317, —
	Period	0–4 weeks	1	0.298 (–0.424 to 1.019)	—	0.568	—	—
	Period	4–8 weeks	3	–7.497 (–13.386 to 0.608)	<.001, 98.5%^d^	—	Moderate	0.117, .02
	Period	8–12 weeks	4	2.130 (–1.520 to 5.781)	<.001, 98.0%^d^	—	Moderate	0.042, .02
	Period	More than 12 weeks	1	*0.381 (0.061-0.701)*	—	—	—	—
**Working memory**								
	Overall	N/A	12	*1.034 (0.305-1.763)*	<.001, 92.3%^d^		Moderate	0.501, .38
	Intervention type	Nintendo Wii	1	0.559 (–0.361 to 1.479)	—	—	—	—
	Intervention type	Other video games	11	*1.076 (0.295-1.858)*	<.001, 93.0%^d^		Moderate	0.139, .17
	Control type	Activity control	8	1.045 (–0.012 to 2.102)	<.001, 94.2%^d^	0.985	Moderate	0.322, .28
	Control type	No game play	4	*1.023 (0.133-1.914)*	<.001, 85.4%^d^	—	Moderate	0.497, .82
	Period	0-4 weeks	2	1.885 (–1.713 to 5.482)	<.001, 98.2%^d^	0.660	Low	0.317, —
	Period	4-8 weeks	3	*1.401 (0.559-2.243)*	.01, 79.4%^d^	—	Moderate	0.602, .86
	Period	8-12 weeks	4	0.016 (–0.841 to 0.874)	.002, 79.9%^d^	—	Moderate	1.000, .94
	Period	More than 12 weeks	2	1.961 (–1.186 to 5.107)	.002, 79.9%^d^	—	Low	0.317, —
**Corsi block test**								
	Overall (all other video games)	N/A	5	1.120 (–0.077 to 2.316)	<.001, 92.3%^d^	—	—	0.221, .38
	Control type	Activity control	4	1.137 (–0.352 to 2.627)	<.001, 94.2%^d^	0.971	Moderate	0.174, .44
	Control type	No game play	1	*1.064 (0.185-1.944)*	—	—	—	—
	Period	0–4 weeks	1	*3.738 (2.883-4.594)*	—	0.006^f^	—	0.497, .54
	Period	8–12 weeks	4	*0.429 (0.080-0.778)*	.42, 0.0%	—	High	—

^a^OR: odds ratio.

^b^TMT: trail-making test.

^c^N/A: not applicable.

^d^Substantial heterogeneity.

^e^—: not applicable.

^f^Sources of heterogeneity.

Regarding general cognition, the first item assessed was the MMSE score. A significant difference was found with Xbox 360 as the intervention type and 4-8 weeks as the intervention period. The second item evaluated was the MoCA score. A significant difference was only found for activity control as the control type (OR 1.826, 95% CI 0.043-3.609), with substantial heterogeneity (Table 5). With respect to physical function, first, in the everyday function outcome, a significant difference was found only in the intervention period of 4-8 weeks (OR –1.045, 95% CI –1.866 to –0.223), with substantial heterogeneity. A significant difference was found in the function test (cm) outcome (OR 0.725, 95% CI 0.235-1.214), with low heterogeneity (*P*=.26, I^2^=25.5%). No significant difference was found for the function test (s) outcome (Table 6). In summary, video game intervention may have an effect on improving general cognitive function, but it had little effect on physical function.

We considered processing speed after video game intervention versus nonvideo game control. First, when we evaluated the TMT-A (s), significant differences were found for overall outcomes (OR –0.833, 95% CI –1.463 to –0.204), other video games (OR –0.874, 95% CI –1.558 to –0.190), activity control (OR –1.033, 95% CI –1.830 to –0.235), and for more than 12 weeks (OR –2.395, 95% CI –4.272 to –0.519), with substantial heterogeneity. In terms of processing speed (number), significant differences were found overall and in subgroups, while substantial heterogeneity was found overall (OR 1.084, 95% CI 0.765-1.402, *P*<.001, I^2^=95.5%) and for activity control (Table 7). In Falls Efficacy Scale assessment, no significant differences were found overall and in all subgroups. In the Geriatric Depression Scale assessment, significant differences were found for Xbox 360 and other video games (OR –0.651, 95% CI –1.164 to –0.138) as the intervention types and 0-4 and 8-12 weeks as the intervention periods (OR –1.800, 95% CI –2.745 to –0.854), with substantial heterogeneity (Table 8). In summary, video game intervention had a significant advantage in terms of processing speed and had a tendency to reduce depression scores.

**Table 5:** Summarized outcomes from pairwise meta-analysis of performance and cognitive data for general cognition (italics indicate a significant difference).

Variable		Subgroup	Studies (n)	OR^a^ (95% CI)	*P* value, I^2^	Meta-regression	Grade	Publication bias, *P* value
**MMSE^b^ (score)**								
	Overall (all activity control)	N/A^c^	3	1.557 (–0.459 to 3.572)	<.001, 95.5%^d^	—^e^	Low	0.296, .37
	Intervention type	Xbox 360	1	*4.606 (3.366-5.846)*	—	0.095^f^	—	—
	Intervention type	Other video games	2	0.215 (–0.175 to 0.605)	.60, 0.0%	—	Moderate	0.317, —
	Period	0–4 weeks	2	0.215 (–0.175 to 0.605)	.60, 0.0%	0.095^f^	Moderate	0.317, —
	Period	4–8 weeks	1	*4.606 (3.366-5.846)*	—	—	—	—
**MoCA^g^ (score)**								
	Overall	N/A	5	1.296 (–0.102 to 2.693)	<.001, 93.9%^d^	—	Moderate	0.624, .01
	Intervention type	Nintendo Wii	1	–0.416 (–1.140 to 0.308)	—	0.419	—	—
	Intervention type	Xbox 360	2	3.796 (–4.023 to 11.616)	<.001, 98.4%^d^	—	Low	0.317, —
	Intervention type	Other video games	2	0.442 (–0.052 to 0.936)	.99, 0.0%	—	Moderate	0.317, —
	Control type	Activity control	4	*1.826 (0.043-3.609)*	<.001, 95.1%^d^	0.594	Moderate	0.317, —
	Control type	No game play	1	–0.416 (–1.140 to 0.308)	—	—	—	—
	Period	0-4 weeks	1	–0.139 (–0.717 to 0.440)	—	0.649	—	0.734, .04
	Period	4-8 weeks	4	1.800 (–0.126 to 3.727)	<.001, 95.2%^d^	—	Moderate	—

^a^OR: odds ratio.

^b^MMSE: Mini-Mental State Exam.

^c^N/A: not applicable.

^d^Substantial heterogeneity.

^e^—: not applicable.

^f^Sources of heterogeneity.

^g^MoCA: Montreal Cognitive Assessment.

**Table 6:** Summarized outcomes from pairwise meta-analysis of performance and cognitive data for physical function (italics indicate a significant difference).

Variable		Subgroup	Studies (n)	OR^a^ (95% CI)	*P* value, I^2^	Meta-regression	Grade	Publication bias, *P* value
**Everyday function**								
	Overall	N/A^b^	12	–0.014 (–0.538 to 0.510)	<.001, 86.3%^c^	—^d^	Moderate	0.837, .90
	Intervention type	Nintendo Wii	3	–1.024 (–2.645 to 0.597)	<.001, 87.3%^c^	0.113	Low	0.602, .94
	Intervention type	Xbox 360	1	–0.016 (–0.594 to 0.562)	—	—	—	—
	Intervention type	Other video games	8	0.306 (–0.283 to 0.895)	<.001, 86.1%^c^	—	Moderate	0.322, .41
	Control type	Activity control	9	0.138 (–0.418 to 0.695)	<.001, 83.9%^c^	—	Moderate	0.532, .64
	Control type	No game play	3	–0.544 (–2.187 to 1.098)	<.001, 93.3%^c^	—	Low	0.602, .20
	Period	0-4 weeks	2	0.568 (–0.634 to 1.769)	.01, 83.6%^c^	0.760	Low	0.317, —
	Period	4-8 weeks	4	–*1.045 (–1.866* to *0.223)*	.003, 78.8%^c^	—	Moderate	0.042, .01
	Period	8-12 weeks	3	1.076 (–0.125 to 2.277)	<.001, 88.3%^c^	—	Low	0.117, .14
	Period	More than 12 weeks	3	–0.035 (–0.324 to 0.255)	.61, 0.0%	—	Moderate	0.117, .62
**Function test (s)**								
	Overall	N/A	4	–0.284 (–1.735 to 1.168)	<.001, 95.3%^c^	—	Moderate	0.308, .22
**Function test (cm)**								
	Overall	N/A	3	*0.725 (0.235-1.214)*	0.26, 25.5%	—	Moderate	1.000, .85

^a^OR: odds ratio.

^b^N/A: not applicable.

^c^Substantial heterogeneity.

^d^—: not applicable.

**Table 7:** Summarized outcomes from pairwise meta-analysis of performance and cognitive data for processing speed (italics indicate a significant difference).

Variable		Subgroup	Studies (n)	OR^a^ (95% CI)	*P* value, I^2^	Meta-regression	Grade	Publication bias, *P* value
**TMT-A^b^ (s)**								
	Overall	N/A^c^	11	–0.833 (–1.463 to *–0.204)*	<.001, 93.0%^d^	—^e^	Moderate	0.020, .01
	Intervention type	Xbox 360	1	–0.531 (–1.180 to 0.117)	—	0.838	—	—
	Intervention type	Other video games	10	–0.874 (–1.558 to *–0.190)*	<.001, 93.6%^d^	—	Moderate	0.009, .01
	Control type	Activity control	9	–1.033 (–1.830 to *–0.235)*	<.001, 94.4%^d^	0.551	Moderate	0.007, .01
	Control type	No game play	2	–0.163 (–0.516 to 0.189)	.87, 0.0%	—	Moderate	0.317, —
	Period	4–8 weeks	4	–0.272 (–0.616 to 0.072)	.49, 0.0%	0.086^f^	High	0.497, .85
	Period	8–12 weeks	3	0.132 (–0.133 to 0.398)	.53, 0.0%	—	Moderate	0.117, .40
	Period	More than 12 weeks	4	–*2.395 (–4.272* to *0.519)*	<.001, 97.7%^d^	—	Moderate	0.042, .004
**Processing speed (number)**								
	Overall (all other video games)	N/A	7	*1.084 (0.765-1.402)*	<.001, 95.5%^d^	—	Moderate	0.096, <.001
	Control type	Activity control	5	*1.374 (0.960-1.789)*	<.001, 96.9%^d^	0.413	Moderate	0.142, .001
	Control type	No game play	2	*0.665 (0.168-1.163)*	.42, 0.0%	—	Moderate	0.317, —
	Period	0–4 weeks	1	*0.842 (0.091-1.592)*	—	0.024^f^	—	—
	Period	8–12 weeks	4	*0.536 (0.170-0.903)*	.74, 0.0%	—	High	0.497, .55
	Period	More than 12 weeks	2	*8.169 (6.916-9.423)*	.94,0.0%	—	Moderate	0.317, —

^a^OR: odds ratio.

^b^TMT: trail-making test.

^c^N/A: not applicable.

^d^Substantial heterogeneity.

^e^—: not applicable.

^f^Sources of heterogeneity.

**Table 8:** Summarized outcomes from pairwise meta-analysis of performance and cognitive data for fear of falling and depression (italics indicate a significant difference).

Variable		Subgroup	Studies (n)	OR^a^ (95% CI)	*P* value, I^2^	Meta-regression	Grade	Publication bias, *P* value
**Falls Efficacy Scale International (score)**								
	Overall	N/A^b^	7	–0.990 (–3.003 to 1.022)	<.001, 99.2%^c^	0.628	—^d^	0.776, .11
	Intervention type	Nintendo Wii	4	–1.305 (–4.232 to 1.621)	<.001, 99.4%^c^	0.628	Moderate	1.000, .04
	Intervention type	Other video games	3	–0.539 (–1.651 to 0.574)	<.001, 87.7%^c^	—	Low	0.602, .50
	Control type	Activity control	4	–0.450 (–1.143 to 0.242)	.001, 81.9%^c^	0.446	Moderate	0.497, .28
	Control type	No game play	3	–1.641 (–5.240 to 1.959)	<.001, 99.4%^c^	—	Low	0.602, .20
	Period	4–8 weeks	5	–0.314 (–0.858 to 0.229)	.003, 75.6%^c^	0.156	Moderate	0.327, .26
	Period	8–12 weeks	2	–2.534 (–6.909 to 1.841)	<.001, 99.6%^c^	—	Low	0.317, —
**Geriatric Depression Scale (score)**								
	Overall	N/A	10	–0.393 (–1.058 to 0.273)	<.001, 91.5%^c^	—	—	1.000, .41
	Intervention type	Nintendo Wii	4	0.683 (–0.797 to 2.164)	<.001, 94.8%^c^	0.876	Moderate	0.174, .02
	Intervention type	Xbox 360	1	–*2.742 (–3.521* to *1.962)*	—	—	—	—
	Intervention type	Other video games	5	–*0.651 (–1.164* to *0.138)*	.004, 74.0%^c^	—	Moderate	0.142, .43
	Control type	Activity control	5	0.135 (–0.941 to 1.211)	<.001, 93.3%^c^	0.352	Moderate	0.142, .07
	Control type	No game play	5	–0.866 (–1.825 to 0.094)	<.001, 91.3%^c^	—	Moderate	0.050, .34
	Period	0–4 weeks	1	–*0.637 (–1.111* to *0.163)*	—	0.403	—	—
	Period	4–8 weeks	5	0.552 (–0.604 to 1.708)	<.001,93.3%^c^	—	Moderate	0.327, .004
	Period	8–12 weeks	3	–*1.800 (–2.745* to *0.854)*	.01,79.1%^c^	—	Moderate	0.117, .35
	Period	More than 12 weeks	1	–0.289 (–0.705 to 0.127)	—	—	—	—

^a^OR: odds ratio.

^b^N/A: not applicable.

^c^Substantial heterogeneity.

### Outcomes of Network Meta-analysis

**Figure 2:**
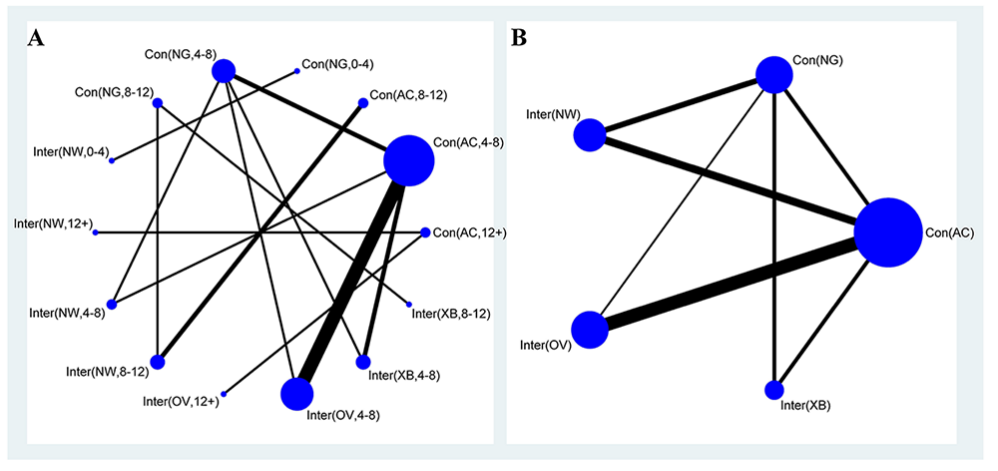
Network plot for all interventions and controls for balance time with (A) and without (B) the intervention period in older adults. Each circular node represents an intervention/control type. The circle size is proportional to the total number of participants, while the line width is proportional to the number of studies used in the head-to-head comparisons. AC: activity control; NG: no game play; NW: Nintendo Wii; OV: other video games; XB: Xbox.

Using pairwise meta-analysis, we observed that the video game intervention improved clinical performance and cognitive function, especially processing speed and depression scores, in the elderly. However, the most suitable video game intervention has not been determined. Moreover, many subgroups included fewer studies, which may have affected the accuracy of the results. Therefore, we selected the outcomes of the included studies to conduct Bayesian network meta-analysis among the 6 indicators to identify the most suitable type of video game intervention for the elderly. Figure 2 provides network plots of balance time with the intervention period (Figure 2A) and without the intervention period (Figure 2B), because most studies were included in this outcome.

For the indicator of balance function, first, in terms of balance time (s), compared with no game play as the control group, Xbox 360 as the intervention type ranked first, with a significant difference (SMD –3.34, 95% CrI –5.54 to –2.56), followed by other video games (SMD –1.15, 95% CrI –2.69 to –0.64), Nintendo Wii, and activity control. Significant differences could also be found in Xbox 360 versus other video games as the intervention type (SMD –1.62, 95% CrI –4.89 to –1.03), Nintendo Wii (SMD –4.14, 95% CrI –9.64 to –0.28), and activity control. For the BBS, significant differences were found only in other video games compared with Nintendo Wii as the intervention type (SMD 0.38, 95% CrI 0.03-1.79), with some publication bias (Multimedia Appendix 4). In summary, the intervention typical of Xbox 360 and other video games may be the best intervention method to maintain balance function over time (Figure 2A).

For general cognition, we combined the scores of MMSE and MoCA. Compared with no game play as the control type, which ranked the lowest, other video games as the intervention type ranked the highest, with a significant difference (SMD 1.23, 95% CrI 0.82-1.86), followed by Xbox 360 (SMD 1.13, 95% CrI 0.74-1.72), Nintendo Wii (SMD 0.90, 95% CrI 0.59–1.37), and activity control (SMD 1.44, 95% CrI 0.94-2.20). Significant differences were found in all comparisons, which suggests that the ordering from our Bayesian network meta-analysis was reasonable. No significant differences were found among the network outcomes from the TMT-B (s) to determine executive function (Figure 2B). In summary, video game intervention improved the cognitive function of elderly patients but had no effect on executive function.

**Figure 3:**
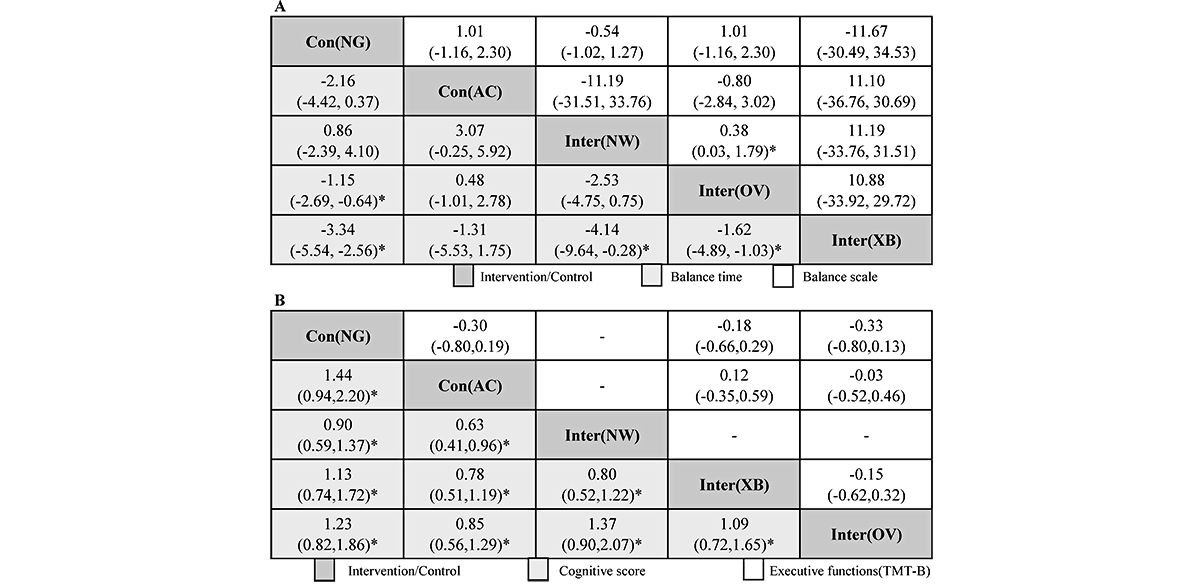
Summary effects from Bayesian network meta-analysis for balance time and BBS (A) and cognitive score and executive function (B) are ranked by the mean rank and the SUCRA score. Information relating to the SMDs and 95% CrIs is listed in the columns, with the rows displaying the intervention identity. SMD values higher than 0 favor the column-defining intervention (ie, the left-most in order), indicating improvement in effectiveness. *Statistical significance. AC: activity control; BBS: Berg Balance Scale; CrI: credible interval; NG: no game play; NW: Nintendo Wii; OV: other video games; SMD: standardized mean difference; SUCRA: surface under the cumulative ranking curve; trail-making test; XB: Xbox.

In the assessment of processing speed, the first item examined was the TMT-A (s). Compared with no game play, other video games ranked first, with a significant difference (SMD –0.29, 95% CrI –0.49 to –0.08), followed by activity control (SMD –0.36, 95% CrI –0.57 to –0.15), virtual reality–based games, and Xbox 360. Significant differences were also found in comparisons of other video games and activity control versus virtual reality–based games and Xbox 360. The second item assessed was processing speed (number). Other video games as the intervention type also ranked first, with a significant difference compared with no game play as the control (SMD 0.72, 95% CrI 0.36-1.09), followed by virtual reality–based games (SMD 0.60, 95% CrI 0.26-0.94) and activity control (SMD 0.42, 95% CrI 0.06-0.77). In general, video game intervention improved processing speed (Figure 3A). For the evaluation of depression scales, compared with no game play, Xbox 360 ranked the highest, followed by other video games, activity control, and Nintendo Wii, with no significant difference. In terms of the Falls Efficacy Scale, there was no significant difference (Figure 3B).

In conclusion, based on Bayesian network meta-analysis, we determined that video game intervention improves balance function, cognitive function, and processing speed, which were similar results to those obtained using pairwise meta-analysis.

## Discussion

### Principal Findings

Our Bayesian network meta-analysis quantified the comparative effectiveness of video games based on 47 studies including 3244 elderly participants, with acceptable quality. We comprehensively summarized the comparative efficacy of video games in improving performance and cognitive function in 6 domains: balance function, executive function, general cognition, physical function, processing speed, and fear of falling and depression. The results suggested that, first, on pairwise meta-analysis, video game interventions are beneficial for cognition scores, processing speed, and depression scores. There are tendencies toward benefits for balance function, executive function, and physical function. Second, on Bayesian network meta-analysis, interventions with video games may improve balance function, cognitive scores, and processing speed in the elderly, which was similar to the results of pairwise meta-analysis. Third, from the ranking of the Bayesian network meta-analysis, Xbox 360 and other video games always ranked first, while Nintendo Wii always ranked last of all interventions. This was accompanied by having the most outcomes of moderate GRADE with low publication bias in both pairwise and Bayesian network meta-analyses.

**Figure 4:**
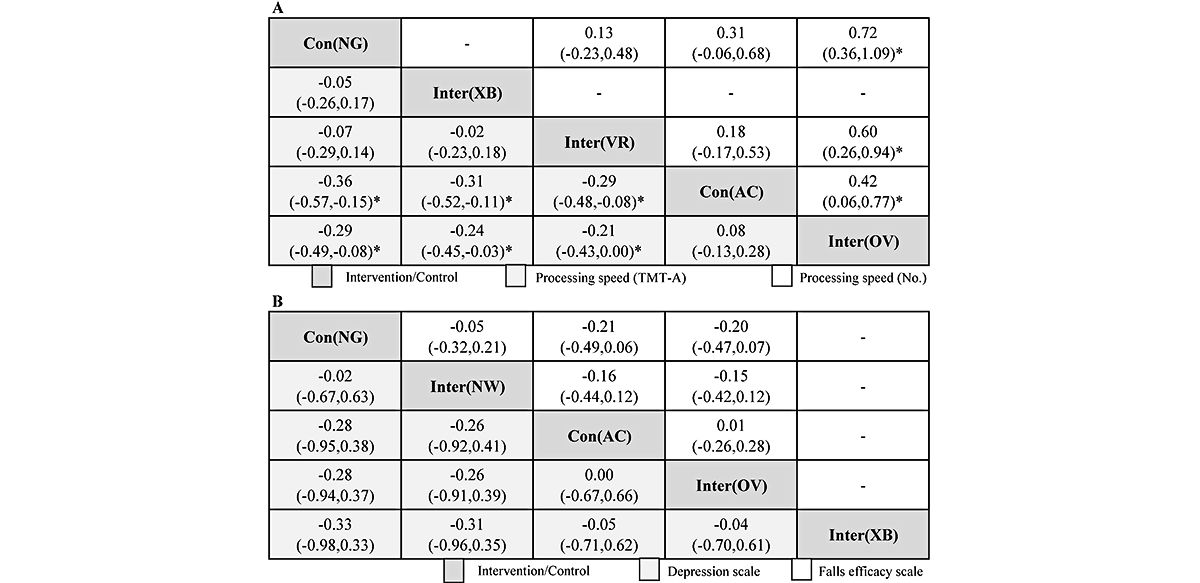
Summary effects from Bayesian network meta-analysis for processing speed (A) and depression and falls efficacy (B) are ranked by the mean rank and the SUCRA score. Information relating to the SMDs and 95% CrIs is listed in the columns, with the rows displaying the intervention identity. SMD values higher than 0 favor the column-defining intervention (ie, the left-most in order), indicating improvement in effectiveness. *Statistical significance. AC: activity control; CrI: credible interval; NG: no game play; NW: Nintendo Wii; OV: other video games; SMD: standardized mean difference; SUCRA: surface under the cumulative ranking curve; trail-making test; XB: Xbox.

In this systematic review, we used a comprehensive search with clear inclusion and exclusion criteria and carefully examined the efficacy of video game interventions in improving performance and cognitive function in 6 domains and 18 outcomes. Generally, the consistency of Bayesian network meta-analysis was similar to that of pairwise meta-analysis. On Bayesian network meta-analysis, compared with no game play as the control type, significant differences were found in balance time (s), cognitive scores, processing speed (TMT-A), and processing speed (number). For expert balance time (s), significant differences were also found in terms of cognitive scores, processing speed (TMT-A), and processing speed (number) on pairwise meta-analysis (Table 2; Figures 3 and 4). Based on the results of our study, video game interventions had the most obvious benefit for cognitive scores and processing speed. Processing speed is defined as the time spent completing mental tasks. It relates to the patient’s speed of understanding the information they obtain, whether it is visual (letters and numbers), auditory (language), or mobile. Similar results were found by Ozdogar et al [67], suggesting that video-based exergaming is almost as effective as conventional rehabilitation with respect to improving walking, upper- and lower-extremity functions, cognitive function, fatigue, depression, and health-related quality of life. Shin et al [68] found that participants who frequently played video games showed enhanced processing speed, which could be an effect of game practice. Mansor et al [2] determined limited effects of video games on cognitive function, and another valuable research published by Wang et al [69] also proved that game-based brain training can be considered a supplementary intervention for improving cognitive function in community-dwelling older adults. Moreover, Vázquez et al’s [70] research indicates that video game–based interventions may assist adults in active aging processes and prevent secondary aging. The above valuable studies all support our results.

In our study, we found that other video games and Xbox 360 are more effective than Nintendo Wii. Other types of video games were defined as exergames, video games, computer-based games, and virtual reality–based games. The main reason for these results may be that Xbox 360 and other video game screens require equipment to play, which have good platforms, a strong visual sense, and good interactivity. However, they are not easy to carry because of the requirement of external equipment.

A previous meta-analysis did not distinguish between different video games; therefore, this is an innovation of our study. A previous study suggested that after Nintendo Wii therapy, patients experienced motor learning retention, achieving a sustained benefit using the technique [71]. We observed that Nintendo Wii only benefited patients in general; therefore, when we choose video game interventions for elderly participants, we should choose better-intervention video game types with better interaction and visual stimulation for older adults. Video games could also improve mental health among older adults [72].

The mechanisms underlying changes following video game interventions remain unclear, although they might be related to tonic/phasic activation or inhibition of affected brain regions during video game playing [73] that make the participants feel satisfied. Specifically, psychological satisfaction and pleasure might be related to the various feedback mechanisms provided to the player by the active video game. This elaborate reinforcement and reward schedule has the potential to maximize motivation [74]. Video games are able to maintain flexibility of striatal responses to reward, a mechanism that might be extremely important to keep motivation high and therefore might be of critical value for many different applications, including cognitive training and therapeutic possibilities [75]. In the studies we included, only a few participants felt fatigue or leg muscle soreness; however, the players could tolerate and relieve themselves, suggesting that video game interventions are safe. Dankbaar et al [76] showed that video lectures from a serious game are effective for specific topics, such as patient safety.

### Limitations

This study had several limitations. First, we predesigned the inclusion criteria of video game intervention versus nonvideo game control. The definition of “video game” was broad, including Xbox 360, Nintendo Wii, virtual reality–based games, and computer-based games. However, the degree of stimulation, interaction, and pleasure of different video games for participants differed, resulting in clinical heterogeneity. Second, substantial heterogeneity was frequently determined on pairwise meta-analysis. One reason is the existence of clinical heterogeneity (many subgroups included less than 3 studies), and the other is the different types of combined scales for outcomes, leading to methodological heterogeneity. Third, some publication bias was detected in both pairwise and Bayesian network meta-analyses, which may be due to the difficulty in publishing negative outcomes. Lastly, to partially maintain the slight stability of the results under the Bayesian framework, we only chose outcomes that included more studies. Therefore, although the Bayesian network meta-analysis was not comprehensive, the results were more accurate.

### Conclusion

In summary, impaired cognitive function is a highly prevalent condition that can profoundly influence the quality of life and accounts for major health care expenditures among the elderly. Our comprehensive Bayesian network meta-analysis provided evidence that video game interventions could be considered for the elderly to improve their performance and cognitive function, especially general cognitive scores and processing speed. Video games with better interactivity and visual stimulation have better curative effects. Based on the available evidence, we recommend video game interventions for the elderly. Future studies should be designed as multicenter RCTs, involving more subjects and providing more detailed description of the types of video games, in order to determine the most appropriate type of video game for older adults.

## Appendix

Multimedia Appendix 1 Baseline characteristics of 47 studies.

Multimedia Appendix 2 Risk-of-bias summary.

Multimedia Appendix 3 Risk-of-bias assessment using the NOS for CCTs. CCT: case-controlled trial; NOS: Newcastle-Ottawa Scale.

Multimedia Appendix 4 Net funnel of balance time.

## Abbreviations

BBS: Berg Balance Scale
BMI: body mass index
CCT: case-controlled trial
CrI: credible interval
GRADE: Grading of Recommendations Assessment, Development and Evaluation
MMSE: Mini-Mental State Exam
MoCA: Montreal Cognitive Assessment
NOS: Newcastle-Ottawa Scale
OR: odds ratio
PRISMA: Preferred Reporting Items for Systematic Reviews and Meta-Analysis
RCT: randomized controlled trial
SMD: standardized mean difference
SUCRA: surface under the cumulative ranking curve
TMT: trail-making test
