# Supplementary material for: The Effect of Video Game–Based Interventions on Performance and Cognitive Function in Older Adults: Bayesian Network Meta-analysis
Source: JMIR Serious Games. 2021 Dec 30;9(4):e27058. doi: 10.2196/27058 (PMC8759017; doi:10.2196/27058)
Supplement: Multimedia Appendix 1 [file games_v9i4e27058_app1.doc]

**Table S1 Baseline characteristic of 47 studies**

| **Study, year** | **Study types** | **Region** | **Sample size(male/female)** | **Age** | **BMI** | **Education** | **MMSE** | **Video game type** | **Frequency** | **Period** | **Follow-up** | **Control type** | **Other associated diseases** | **Community or hospital** |
| --- | --- | --- | --- | --- | --- | --- | --- | --- | --- | --- | --- | --- | --- | --- |
| Rica RL, 2020[1] | RCT | Brazil | I:25  C:25 |  | I:28±5  C:28±5 |  |  | X-BOX 360 Console | 60-min sessions, three times a week | 3 months |  | no-game-play | None | Hospital |
| Amjad I, 2019[2] | RCT | Pakistan | I:20  C:18 | - |  |  |  | Xbox 360 with Kinect | 25 to 30  minutes, 5 days a week | 6 weeks | - | normal joint range of motion and stretching exercises of upper and lower limbs | mild cognitive impairment (MCI). | Hospital |
| Belchior P, 2019[3] | RCT | Canada | I:17  C(a):19  C(b):18 | 73.2 ±5.5; 20/34 | C：31.18 ± 2.5  I：  28.02 ± 1.67 |  |  | Videogame training (using the off-the-shelf driving game,  Crazy Taxi [CT]) | 5 hours a week, 1 hour a day | 3 months | 3 months | C(a): Insight process-based  intervention  C(b):no trained groups | None | Community |
| Faust ME, 2019[4] | RCT | USA | I:25  C:19 | I: 78.0±6.6, 9/16  C:79.8±5.4.8/11 |  |  |  | computer-disk games | 30-40 training sessions of 30-  40 min each in 8-10-weeks | 8-10 weeks | 3 months | no-game-play | None | Community |
| Gatica-Rojas V, 2019[5] | CCT | Chile | I:7  C:5 | I:69.14 ±5.8, 2/5  C:69 ± 3.67. 2/3 |  | C：31.18 ± 2.5  I：  28.02 ± vv |  | Nintendo Wii balance board (NWBB) | 25 minutes/session,18 sessions | 6 weeks | - | Tai Chi Chuan | None | Community |
| Liao YY, 2019[6] | RCT | China | I:18  C:16 | I:75.5 ± 5.2, 7/11  C:73.1 ± 6.8.4/12 | I： 24.4±4.2  C：  23.2±3.6 | I：9.3±3.8  C:  9.9±2.1 | I：  22.84±2.69  C;  23.15±2.96 | Virtual Reality-Based Physical and Cognitive Training | 60-min, three times a week | 12 weeks | - | Combined Physical and Cognitive Training | None | day care  centers |
| Montero-Alía P, 2019[7] | RCT | Spain | I:508  C:469 | I:75.1 ±1.56  C:75.4 ±1.51 | I：  27.8 ±1..35  C:  27.5 ±1.53 | - |  | Nintendo WiiFit^TM^ game | Two  30-min sessions per week | 3 months | - | no-game-play | None | Health care  centres |
| Perrot A, 2019[8] | RCT | France | I:12  C(a):12  C(b):11 | I:64.37±3.17  C(a):63.75±2.49  C(b):65.55±2.91 |  | I:  11.91±2.84  C（a）：  12.00±2.92  C（b）：  11.36±3.55 |  | Video game: Super Mario Bros  examgame | 1 hour, thrice per week | 8–10 weeks | - | C(a): Kawashima Brain Training  C(b):no-training no-contact control group | None | Community |
| Sápi M, 2019[9] | RCT | Hungary | I:30(1/29)  C(a):23(1/22)  C(a):22(4/18) | I:69.57± 4.66  C(a):69.12± 4.19  C(a):67.18 ± 5.56 | I ： 25.95±2.60  C；  27.09±5.45 |  | I：25.95±2.60  C: 27.095.45 | Microsoft for Xbox 360 video game | 30 minutes , three times a  week | 6 weeks | - | C(a): Adventures and Sports  C(b):no-game-play | None | Community |
| Sosa GW, 2019[10] | RCT | USA | I:20(4/16)  C:15(5/10) | I:74.95 ±6.53  C:74.40 ±5.62 |  |  |  | Video game | one-hour sessions per week | 5 weeks | 12 weeks | no-game-play | None | Community |
| Zadro JR, 2019[11] | RCT | Australia | I:30(12/18)  C:30(17/13) | I:68.8±5.5  C:67.8±6.0 | I：26.9 ±4.1 C:27.4 ±3.6 |  |  | Nintendo Wii U console with Wii Fit U software | 60 minutes, 3 times per week | 8 weeks | 3 months, and 6 months | no-game-play | None | Community |
| Bacha JMR, 2018[12] | RCT | Brazil | I:23(8/15)  C:23(4/19) | I:71.0 ±2.16  C:66.5 ±1.72 | I：24.96 ±1.27  C: 24.82 ±1.32 |  |  | Xbox Kinect Adventures games | two sessions a week, 60-minute duration each intervention | 4 weeks | 30 days | Conventional Physical Therapy | None | Community |
| Gomes GCV, 2018[13] | RCT | Brazil | I:15  C:15 | I:83±5.87  C:85±6.19 |  |  |  | Nintendo Wii Fit Plus | 50 min, twice a week | 14 sessions ,7 weeks | 30 days | no-exercise control group | frail older adult | Hospital |
| Li J, 2018[14] | RCT | Singapore | I:49(20/29)  C:53(17/36) | I:71.12±8.67  C:71.66±7.13 |  |  |  | digital sport condition was Nintendo’s Wii | once a week, for six weeks | 6 weeks |  | traditional exercise | None | Community |
| Szelag E, 2018[15] | RCT | Spain | I:30  C:25 | I:66.40±5.64  C:64.52±4.51 |  |  | I:28.70 ±1.29  C：  29.00 ±0.91 | 16 sessions with non-action video games from Lumosity, Playing Koi, Highway Hazards, Speed Match, Tidal Treasures, Star Search, Color Mach, Lost in Migration, Pinball Recall, Ebb and Flow, and Disillusion. | 16 training  sessions of ∼40–50 min each | 10–12 weeks | 3 months | same number of sessions with simulation strategy games | None | Community |
| Buitenweg JIV, 2017[16] | RCT | Netherlands | I(a):56(20/36)  I(b):33(13/20)  C:50(23/27) | I(a):64.3  I(b):63.6  C:54 |  | I：5.8 ± 0.7  C：  5.9 ± 0.9 |  | nine games in  three domains: reasoning, working memory, and attention  I(a): frequent switching  I(b):infrequent switching | I(a): one training session consisted of 10 games of 3 min each  I(b):three games of 10 min each were played so that switching between  game domains occurred less frequently. | 12 weeks | 4 weeks | Visual stimulation and feedback and put equal demands on computer ability | None | Community |
| Lee Y, 2017[17] | RCT | Korea | I:21(9/12)  C:19(8/11) | I:76.15± 4.55  C:75.7± 4.91 |  |  |  | The Wii-Fit game, Wii Fit board, and Wii Fit joystick (Nintendo, Japan) were used for the virtual reality training. | 60-min training session, twice a week | 6 weeks |  | fall prevention education | None | Community |
| Monteiro-Junior RS, 2017[18] | RCT | Brazil | I:10(1/9)  C:9(2/7) | I:86± 7  C:86± 5 |  |  | I：22±4  C：25±3 | Nintendo Wii, X-Box, Playstation | 30–45 min of session | - | - | CG performed exactly the same movements as the WG.  However, this group has not received virtual feedback | None | Hospital |
| Ordnung M, 2017[19] | RCT | Germany | I:14(7/7)  C:15(7/8) | I:69.79± 6.34  C:68.6± 4.67 | I:  25.54 ±3.84  C:  26.36 ±4.85 |  |  | exergame training group | Each session lasted 60 min,with 2  sessions per week. | 6 weeks | - | no training group | None | Community |
| Souders DJ, 2017[20] | RCT | USA | I:30(13/17)  C:30(13/17) | I:72.35± 5.20  C:72.27± 4.88 |  |  |  | Western-themed game hub comprised of seven gamified cognitive tasks modified to improve the tasks’ aesthetics | totaling 45 min of  playtime each session. | 1 month |  | playing three common puzzle games each  day | None | participant database |
| Eggenberger P, 2016[21] | RCT | Switzerland | I:19(7/12)  C:14(5/9) | I:72.8±5.9  C:77.8 ±7.4 |  | I:  13.8 ±1.8  C  13.6  ±2.1 |  | cognitive-motor video game dancing | three sessions of 30 min per week | 8-week |  | balance and stretching training | None | Community |
| Kwok BC, 2016[22] | RCT |  | I:40(8/32)  C:40(4/36) | I:69.8 ±7.5  C:70.5 ±6.7 | I:  21.8±3.4  C：  23.3±4.1 |  |  | Nintendo WiiActive gaming exercises with the Wii balance board and resistance band | three sessions of 10 min per week | 12 weeks | 12 weeks | Gym exercise class | None | Hospital |
| Nouchi R, 2016[23] | RCT | USA | I:36  C:36 | I:69:14 ±3:70  C:68:88± 3:73 |  | I：12.44 ±3.49  C：11.82 ±3.43 | I:  (28.36 1.59)  C:  (28.5 1.29) | processing speed training game | 15 min, at least 5 days per | 4 weeks. |  | knowledge quiz training game (active control group ) | None | Community |
| Schättin A, 2016[24] | RCT | USA | I:13(8/5)  C:14(7/7) | I:80±2.55  C:80 ±2.42 |  |  | I:  29±0.25  C:  28.5  ±0.51 | video game-based physical exercise | 30 min sessions three times per week | 24 training sessions, 8 to 10 weeks, |  | conventional balance training | None | Community |
| Toril P, 2016[25] | RCT | Spain | I:19  C:20 | I:69.95±6.73  C:73.20 ±6.48 |  | I  :13.37 ±3.27 C:  12.85 ±3.36 | I:  28.31 (1.00)  C:  27.75 (1.48) | video games | 1-h training sessions, 15 times | 7-8 weeks | 3-month | no training group | None | Community |
| Yeşilyaprak SS, 2016[26] | RCT | Turkey | I:7  C:11 | I:70.1 ± 4.0  C:73.1 ± 4.5 | 27.0 ± 6.6  26.6 ± 4.3 |  |  | Virtual reality game | 35–45 minutes of training, 3 sessions per week | 6-week |  | Conventional exercise | None | Community |
| Eggenberger P, 2015[27] | RCT | netherlands | I:24(10/14)  C(a):22(6/16)  C(b):25(9/16) | I:77.3 ± 6.3  C(a):78.5 ±5.1  C(b):80.8 ±4.7 |  | I:13.7 ±1.5  C（a）13.9 ±2.1  C(b): 12.0 ±2.1 | I： 28.4 ±1.4  C（a）： 28.3 ±1.2  C（b）：  28.0 ±1.7 | virtual reality video game dancing | Two 1-hour training sessions per week | 6-months | 1-year | C(a):Treadmill memory training  C(b):Treadmill walking | None | Community |
| Gschwind YJ, 2015[28] | RCT | Australia | I:78(35/43)  C:75(25/50) | I:74.7 ± 6.7  C:74.7 ± 6.0 | I:  26.1±3.8  C:  26.5±3.9 |  |  | exercise program based on best practice commendations for exercise | three balance sessions of about 40 min, three muscle strength sessions of about 15 to 20 min each | 16-week |  | Education booklet | None | Community |
| Karahan AY, 2015[29] | RCT | Turkey | I:48(27/21)  C:42(24/18) | I:71.3 ± 6.1  C:71.5 ± 4.7 | I:  26.8 ± 4.6  C:  27.2 ± 3.2 |  |  | a game set comprising the Xbox 360 Kinect game | 30 min, 5 days a week | 6 weeks |  | Home exercise group | None | Community |
| Kim KW, 2015[30] | RCT | Korea | I:14  C:14 | 72.3 ±5.1 |  |  |  | Neurocognitive function tests | 40 minutes a day three times a week | 8 weeks |  | typical senior community center-based activities | None | Community |
| Sato K, 2015[31] | RCT | Japan | I:28(6/22)  C:26(5/21) | I:70.07 ±5.35  C:68.50 ±5.47 | I:25.88±3.60)  C:25.72±3.99 |  |  | Kinect and Kinect SDK | 40 minutes to 1 hour, two to three times per week | 24 times |  | no game group | None | Community |
| Schoene D, 2015[32] | RCT | Australia | I:47(16/31)  C:43(14/29) | I:82 ± 7  C:81 ± 7 |  |  |  | intervention group played four stepping games | three 20-minute sessions per week | 16 weeks |  | no game group | None | Community |
| Whyatt C, 2015[33] | RCT | United Kingdom | I:40(5/35)  C:42(20/22) | I:77.18± 6.59  C:76.62± 7.28 |  |  | 28.20±1.36  28.06  ±1.56 | balance game training | 30 minutes per session, two times a week | 5 weeks |  | physical activity | None | Community |
| Ballesteros S, 2014[34] | RCT | Sweden | I:17  C:13 | I:68.8±5.15  C:69.2±5.91 |  | I:  12.2 ±5 .09  C:  12.9  ±3.28 | I:  28.7 ±1 .16  C:  28.8  ±1.03 | web-based cognitive training platform that includes games | 20 1-h training sessions | 10–12 weeks |  | met three times with the researchers in a room of the laboratory | None | Community |
| Belchior P, 2013[35] | RCT | Canada | I:14  C(a):15  C(b):13 | I:74.8 ±6.3  C(a):75.8 ±8.5  C(b):73.7 ±5.3 |  | I:  15.8±2.5  C（a）  15.6±2.6  C（b）  16.5±2.6 |  | Participants played the “first person  shooter” video game Medal of Honor – Rising Sun | - | - |  | C(a):placebo training condition  C(b):no intervention | None | Community |
| Jorgensen MG, 2013[36] | RCT | Denmark | I:28(9/19)  C:30(9/21) | I:75.9±5.7  C:73.7±6.1 | I：  26.4 ± 4.1  C:  25.9 ± 4.2 |  |  | biofeedback-based Nintendo Wii training | 35±5 minutes, 2 times a week | 10 weeks |  | daily use of ethylene vinyl acetate copolymer insoles | None | Community |
| Schoene D, 2013[37] | RCT | Australia | I:15  C:17 | I:77.5±4.5  C:78.4±4.5 |  |  | I:  28.96±1.1  C:  28.86±1.1 | Dance Dance Revolution game Stepmania | 2-3 sessions per week for 15-20 minutes | 8 weeks |  | perform usual activities | None | Community |
| Singh DK, 2013[38] | RCT | Singapore | I:18(0/18)  C:18(0/18) | I:61.12 ± 3.72  C:64.00 ± 5.88 |  |  | I:  29.54 + 5.01  C:  27.31 ±4.31 | balance-focused, interactive, virtualreality games, using the Nintendo® Wii balance board | twice a week for 40 min during | 6 weeks |  | therapeutic balance  exercise group | None | Community |
| Daniel K, 2012[39] | RCT | TX | I:8(3/5)  C(a):8(3/5)  C(b):7(3/4) | I:80± 3.37  C(a):78.13± 5.5  C(b):72.6± 4.6 | I：26.9 ±4.0  C（a）：  24.9 ±3.7  C（b）29.4 ±1.4 |  |  | This group used a Nintendo Wii, utilizing basic games such as bowling, tennis, and boxing. | 45 minutes three times per week | 15 weeks |  | C(a):seated exercise group  C(b):whatever physical activity | None | Community |
| Franco JR, 2012[40] | RCT | USA | I:11(2/9)  C(a):11(3/8)  C(b):10(2/8) | I:79.8 ± 4.7  C(a):77.9 ± 6.9  C(b):76.9 ± 6.3 |  |  |  | received Wii Fit balance training and completed supplemental home exercises | twice a week | three weeks |  | C(a):completed exercises from the MOB Program  C(b):no intervention. | None | Community |
| Maillot P, 2012[41] | RCT | France | I:15  C:15 | I:73.47± 4.10  C:73.47± 3.00 | I:  27.40 ±4.46  C:  27.62 5.89 | I:11.20 ±1.78  C：  11.40± 2.22 | I：  28.67 ±1.17  C：  29.27± 0.88 | Exergame training we used the Nintendo Wii, a videogame console with motion-sensitive technology. | 24, 1 hr of training | 12 weeks |  | no intervention. | None | Community |
| Nouchi R, 2012[42] | RCT | Japan | I:14(6/8)  C:14(7/7) | I:68.86±2.07  C:69.31±2.82 |  | I：13.43 ±2.38  C：  13.36 ±2.13 | I：  28.50 ±1.16  C:  28.50 ±1.51 | video game  training (Brain Age or Tetris) | with 5 training days in  each week | 4 weeks |  | placebo training condition | None | Community |
| Pichierri G, 2012[43] | RCT | Switzerland | I:11(3/8)  C:11(1/10) | I:86.9± 5.1  C:85.6±4.2 |  |  |  | Dance video game group | twice weekly | 12 weeks |  | no intervention. | None | Community |
| Rendon AA, 2012[44] | RCT | UK | I:20  C:20 |  |  |  |  | virtual reality gaming,Wii Fit software package | 35–45 min , 3×/week | 6 weeks |  | Usual care | None | Community |
| Singh DK, 2012[45] | RCT | Malaysia | I:18  C:18 | I:61.12±3.72)  C:64.00 ±5.88 |  |  |  | Nintendo® Wii Fit with a Balance Board was used and Balance Bubble | 40 min, twice a week | 6 weeks |  | no intervention. | None | Community |
| van Muijden J, 2012[46] | RCT | Netherlands | I:53(25/28)  C:19(15/4) | I:67.8±3.8  C:67.2 ±3.4 |  |  |  | videogames were custom built, inspired by commercially available cognitive training games | 30-min intervention session per day, every day of the week, | seven week |  | answered quiz questions about documentaries online | None | Community |
| Szturm T, 2011[47] | RCT | Canada. | I:13(3/10)  C:14(5/9) | I:80.5 ±6  C:81 ±7 |  |  | I:  28±3  C:  28±2 | Interactive Computer Game Exercise Regimen | 45 minutes , 2 sessions per week | 8 weeks |  | typical rehabilitation program currently provided | None | Community |

I: Intervention arm; C: Control arm; RCT: randomized controlled trial; CCT: case-controlled trial;
