# Supplementary material for: The Effect of Video Game–Based Interventions on Performance and Cognitive Function in Older Adults: Bayesian Network Meta-analysis
Source: JMIR Serious Games. 2021 Dec 30;9(4):e27058. doi: 10.2196/27058 (PMC8759017; doi:10.2196/27058)
Supplement: Multimedia Appendix 2 [file games_v9i4e27058_app2.pdf]

	Random sequence generation (selection bias)	Allocation concealment (selection bias)	Blinding of participants and personnel (performance bias)	Blinding of outcome assessment (detection bias)	Incomplete outcome data (attrition bias)	Selective reporting (reporting bias)	Other bias
Amjad I, 2019	+	+	−	+	+	?	?
Bacha JMR, 2018	+	+	−	+	?	+	?
Ballesteros S, 2014	+	+	?	+	?	+	?
Belchior P, 2019	+	+	+	−	?	?	?
Bieryla KA, 2013	+	?	−	−	?	?	+
Buitenweg JIV, 2017	+	+	+	+	+	?	+
Daniel K, 2012	+	?	+	+	?	−	−
Eggenberger P, 2015	+	+	+	+	?	−	?
Eggenberger P, 2016	+	+	+	+	+	?	?
Faust ME, 2019	+	+	+	+	−	−	?
Franco JR, 2012	+	+	+	+	+	+	?
Gomes GCV, 2018	+	+	+	+	+	+	?
Gschwind YJ, 2015	+	+	+	+	+	?	?
Jorgensen MG, 2013	+	+	+	+	+	?	+
Karahan AY, 2015	+	+	−	+	?	?	?
Kim KW, 2015	+	+	−	+	+	?	?
Kwok BC, 2016	+	+	+	+	+	?	+
Lee Y, 2017	+	+	+	+	+	?	?
Liao YY, 2019	+	+	−	+	+	+	?
Li J, 2018	+	+	−	+	+	?	?
Maillot P, 2012	+	+	−	+	+	?	?
Monteiro-Junior RS, 2017	+	+	−	+	+	+	?
Montero-Alía P, 2019	+	+	−	+	?	?	+
Nouchi R, 2012	+	+	+	+	+	?	+
Nouchi R, 2016	+	+	−	?	+	?	?
Ordnung M, 2017	+	+	−	+	?	+	?
Perrot A, 2019	+	+	−	−	?	+	?
Pluchino A, 2012	+	+	−	+	?	?	?
Rendon AA, 2012	+	+	−	+	+	?	?
Rica RL, 2020	+	+	+	?	+	?	?
Sápi M, 2019	+	−	−	+	+	?	+
Sato K, 2015	+	+	?	+	−	−	?
Schättin A, 2016	+	+	−	+	+	?	?
Schoene D, 2013	+	+	−	+	+	?	?
Schoene D, 2015	+	+	−	+	+	?	?
Singh DK, 2012	+	+	+	+	+	+	?
Singh DK, 2013	+	+	+	+	+	+	?
Sosa GW, 2019	+	+	+	+	+	?	+
Souders DJ, 2017	+	+	−	+	+	?	?
Szelag E, 2018	+	+	−	+	?	?	?
Szturm T, 2012	+	+	+	+	+	+	+
Toril P, 2016	+	+	+	+	+	?	+
van Muijden J, 2012	+	+	+	+	+	+	+
Whyatt C, 2015	+	+	?	+	+	+	+
Yeşilyaprak SS, 2016	+	+	+	+	+	+	+
Zadro JR, 2019	+	+	+	+	+	?	?
