# Supplementary material for: The Effect of Video Game–Based Interventions on Performance and Cognitive Function in Older Adults: Bayesian Network Meta-analysis
Source: JMIR Serious Games. 2021 Dec 30;9(4):e27058. doi: 10.2196/27058 (PMC8759017; doi:10.2196/27058)
Supplement: Multimedia Appendix 3 [file games_v9i4e27058_app3.doc]

**Table S2 Risk of Bias Assessment using the Newcastle-Ottawa Scale for Case-control Studies**

| **Publication(year)** | **Is the case definition adequate** | **Representativeness of the cases** | **Selection of Controls** | **Definition of Controls** | **Comparability of cases and controls (/2)** | **Ascertainment of exposure** | **Same method of ascertainment for cases and controls** | **Non-Response rate** | **Overall rating and TOTAL SCORE / 10** |
| --- | --- | --- | --- | --- | --- | --- | --- | --- | --- |
| Gatica-Rojas V, 2019[7] | 1 | 1 | 1 | 0 | 1 | 1 | 1 | 1 | 7 |
