# Supplementary material for: The Effect of Video Game–Based Interventions on Performance and Cognitive Function in Older Adults: Bayesian Network Meta-analysis
Source: JMIR Serious Games. 2021 Dec 30;9(4):e27058. doi: 10.2196/27058 (PMC8759017; doi:10.2196/27058)
Supplement: Multimedia Appendix 4 [file games_v9i4e27058_app4.pdf]

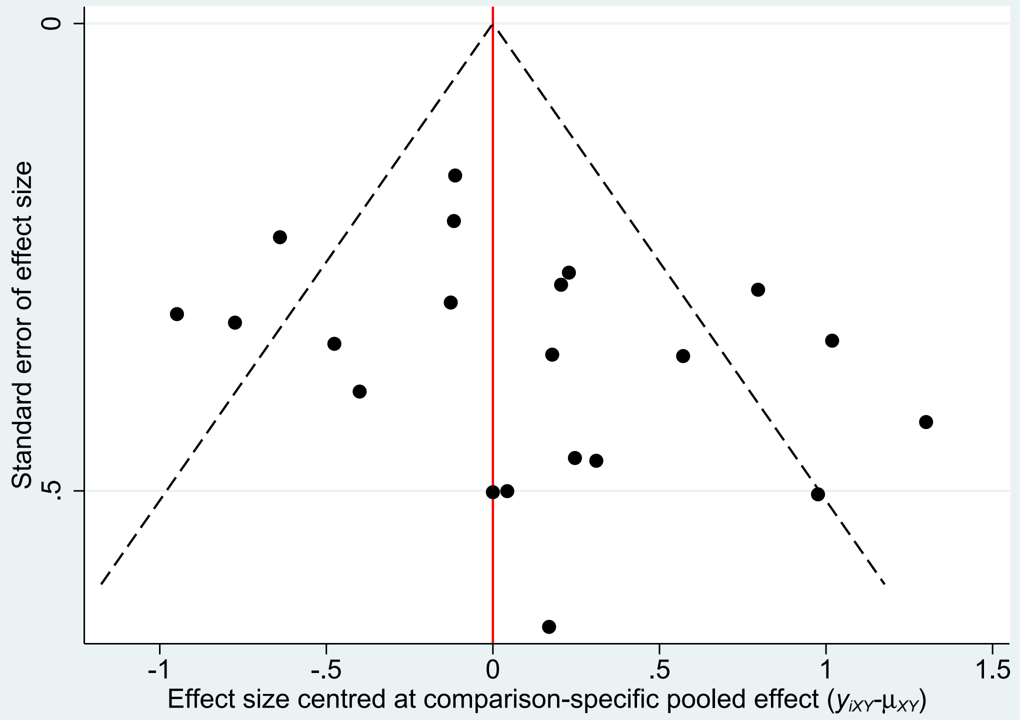

0
.5
Standard error of effect size
-1
-.5
0
.5
1
1.5
Effect size centred at comparison-specific pooled effect (y_{iXY}-μ_{XY})
